# Synchronous OEIC Integrating Receiver for Optically Reconfigurable Gate Arrays

**DOI:** 10.3390/s16060761

**Published:** 2016-05-25

**Authors:** Carlos Sánchez-Azqueta, Bernhard Goll, Santiago Celma, Horst Zimmermann

**Affiliations:** 1 Institute of Electrodynamics, Microwave and Circuit Engineering, Vienna University of Technology, Gußhausstraße 25/354, Vienna 1040, Austria; bernhard.goll@tuwien.ac.at (B.G.); horst.zimmermann@tuwien.ac.at (H.Z.); 2 Group of Electronic Design, Aragón Institute of Engineering Research, Universidad de Zaragoza, Pedro Cerbuna 12, Zaragoza 50009, Spain; scelma@unizar.es

**Keywords:** integrated optoelectronics, integrated *pin* photodiode, integrating receiver

## Abstract

A monolithically integrated optoelectronic receiver with a low-capacitance on-chip *pin* photodiode is presented. The receiver is fabricated in a 0.35 μm opto-CMOS process fed at 3.3 V and due to the highly effective integrated *pin* photodiode it operates at μW. A regenerative latch acting as a sense amplifier leads in addition to a low electrical power consumption. At 400 Mbit/s, sensitivities of −26.0 dBm and −25.5 dBm are achieved, respectively, for *λ* = 635 nm and *λ* = 675 nm (BER = 10^−9^ ) with an energy efficiency of 2 pJ/bit.

## 1. Introduction

Field programmable gate arrays (FPGAs) require reconfiguration times of several milliseconds [1], which makes them unsuitable for applications demanding high-speed reconfiguration capabilities. For such applications, alternative solutions such as multi-context FPGAs, digital application processors with distributed network architectures (DAPs/DNAs) or dynamic reconfigurable processors (DRPs) have been developed, whose operation is based on incorporating several reconfiguration contexts in specific memory banks. Such solutions achieve reconfiguration speeds in the range of hundreds of megahertz but at the cost of a very low gate density.

To address this issue, holographic memories in conjunction with optically reconfigurable gate arrays (ORGAs) are proposed as a viable solution for applications demanding high reconfiguration speeds and gate densities [1,2]. The reconfiguration contexts are stored in the whole 3-D volume of the holographic memory in a page structure, thus achieving high density, and they are retrieved projecting the information stored in each page on an array of optical detectors, which allows operation at hundreds of megahertz [1,2].

The optical detectors in charge of retrieving the information stored in the holographic memory need to satisfy stringent requirements in terms of sensitivity, integration density and low power. As a consequence, solutions based on conventional optical detectors with a transimpedance amplifier (TIA) are not feasible due to their large area and compromise between power consumption and performance. For example, [3] achieves good energy efficiency at 1 pJ/bit but at an input optical average power of −1.7 dBm and in a very expensive 32 nm silicon on insulator (SOI) CMOS process; more recently, an optical detector at 2.7 pJ/bit in 65 nm has been reported [4], but requiring −4.9 dBm average optical power. In contrast, [5] operates at a reduced −31.8 dBm input optical power but with 55 pJ/bit energy efficiency and using a high-voltage avalanche photodiode (PD).

An alternative to TIA-based optical receivers that is gaining attention for communications applications with a tight power budget is the integrating optical receiver. Already introduced for optical memories [6] or highly parallel optical interconnects [7], it mainly uses digital circuitry, which results in a drastic reduction in power consumption and makes it compatible with process scaling to achieve higher data rates. Recently reported implementations achieve Gbit/s with energy efficiencies of 0.9 pJ/bit [8] and 4.5 pJ/bit [9]. In [8] a multi-quantum well (MQW) *pin* PD was flip-chip bonded to a CMOS chip and in [9] an electroabsorption modulator at 1550 nm was used. However, the main disadvantage of integrating optical receivers is their reduced sensitivity, which is reported to be −16.5 dBm at 1 Gbit/s in [8], and −19.4 dBm at 1.2 Gbit/s in [9].

**Figure 1:**
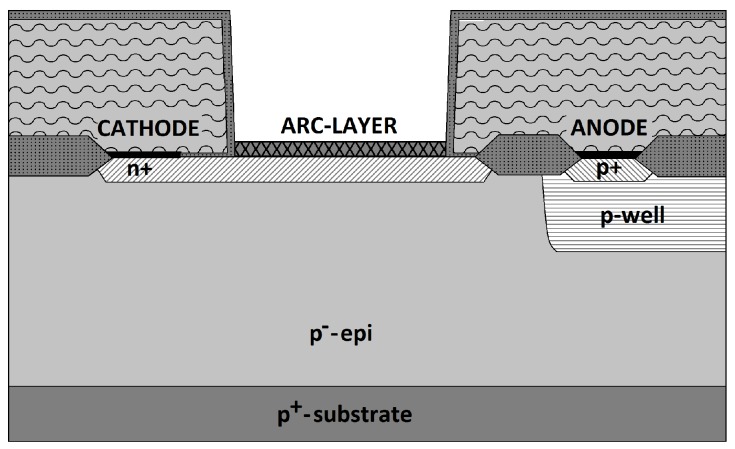
Cross-section of the integrated *pin* photodiode with anti-reflective coating (ARC) layer and low-doped, 15 μm thick epitaxial p- layer.

In an integrating optical receiver, the voltage that is generated by the photocurrent across the PD’s capacitance, and therefore its photo-sensitivity, is inversely proportional to the capacitance value. For this reason, highly sensitive integrating receivers can be implemented by their monolithic integration into an opto-electronic integrated circuit (OEIC) along with the PD. Standard silicon CMOS and BiCMOS technologies allow the integration of small-area *pin* PDs that feature a responsivity higher than 0.5 A W^−1^ and a −3 dB cut-off frequency above 1 Hz with a parasitic capacitance lower than 100 fF [10]. A cross-section of the fabricated *pin* photodiode with anti-reflective coating (ARC) layer is shown in Figure 1. Its bandwidth exceeds 500 MHz already for a reverse bias of −2 V at 675 nm [11]. The low doped (a few 10^13^ cm^−3^), 15 μm thick epitaxial p- layer is responsible for the high bandwidth and the low capacitance of the integrated *pin* photodiode, whereas the ARC layer and the optical window contribute to enhance its responsivity, which would drop by 19% at 650 nm if they were not present.

This paper presents a monolithic integrating optical receiver with a highly efficient 50 μm *pin* PD featuring 50 fF capacitance. The circuit is implemented in a 0.35 μm opto-CMOS process fed at 3.3 V and it achieves 400 Mbit/s with a bit error ratio (BER) better than 10^−9^ for an average input optical power of −26 dBm. The regenerative latch consumes 790 μW, which yields an energy efficiency of 2 pJ/bit, and, along with the output buffer (excluding the PDs) occupies 50 μm × 30 μm.

## 2. OEIC Architecture and Operation

**Figure 2:**
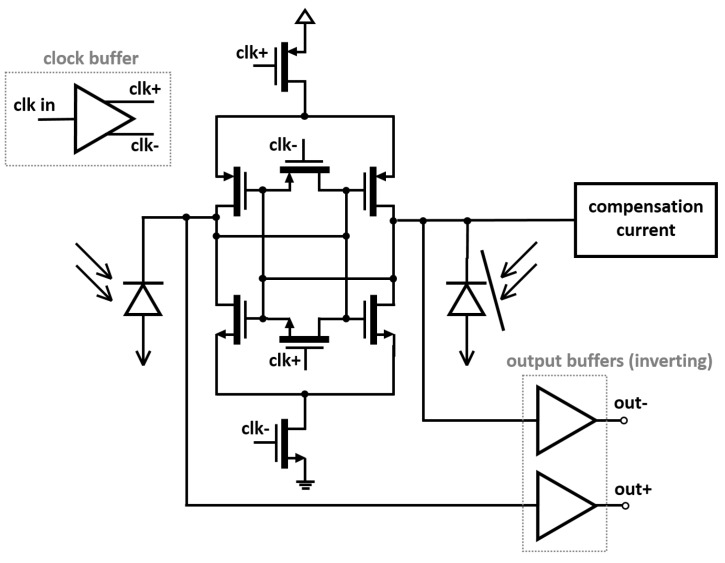
Circuit diagram of the opto-electronic integrated circuit (OEIC). The two clock phases (clk+ and clk−) successively activate and deactivate the latch.

Figure 2 depicts the schematic diagram of the OEIC, which includes a regenerative latch acting as a sense amplifier and two monolithically integrated *pin* PDs, one of which is blocked to the light by a metal layer. A single-ended clock signal, synchronized to the incoming data stream, is driven and converted to a differential clock by two chains of scaled CMOS inverters.

One of the clock phases resets the regenerative latch to a meta-stable state characterized by *V*_*out*+_ = *V*_*out*−_ ≈ *V_dd_*/2 and the other phase allows its operation as a latch. If the incoming data bit corresponds to a logic ‘0’, *i.e.*, no light illuminates the non-blocked PD, the meta-stable state of the latch is broken by a compensation current (Figure 2) discharging the capacitance of the blocked PD, which drives the latch reliably to the stable state (*out*− to ‘1’ and *out*+ to ‘0’, note the inverting behaviour of the buffers). In turn, if the incoming data bit corresponds to a logic ‘1’, *i.e.*, the non-blocked PD is illuminated, the photocurrent generated within discharges its capacitance and drives the latch to the other stable state (*out*− to ‘0’ and *out*+ to ‘1’).

A compensation circuit adding a low capacitance to the latch output node delivers a current to compensate the mismatch of the latch. To minimize the BER, the compensation circuit is controlled from outside manually. Finally, the output signals are driven by a three-stage output buffer formed by an actively loaded common-source stage for impedance matching, two transmission gates triggered by inverted clock signals to obtain an output NRZ signal, and two CMOS inverters to drive the signal to the output bond pads.

## 3. Measurements and Results

**Figure 3:**
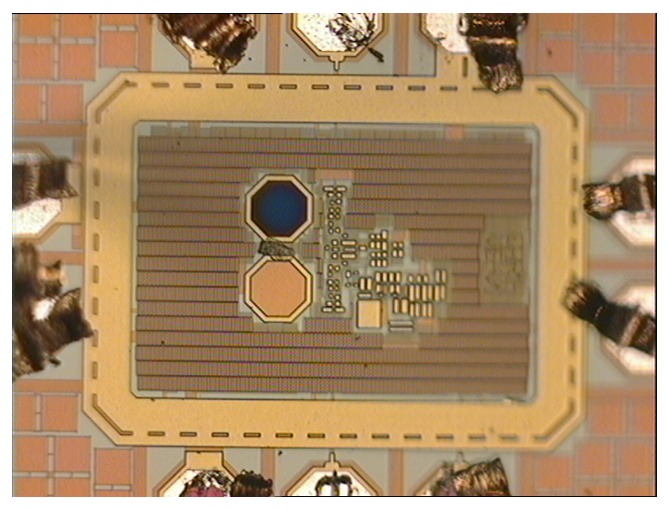
Micro-photograph of the fabricated prototype.

The synchronous integrating receiver is fabricated in a 0.35 μm opto-CMOS process fed at 3.3 V. A micro-photograph of the prototype is shown in Figure 3.

The measurements have been carried out using two different laser sources. The first one is an edge-emitting laser with *λ* = 635 nm and extinction ratio ER = 10, whereas the second one is a vertical cavity surface-emitting laser (VCSEL) with *λ* = 675 nm and ER = 5.5. The two laser sources are then modulated with the pseudo-random bit sequence (PRBS) generated by a Sympuls BMG-2500 bit pattern generator, and the average power of the modulated light signal is controlled by an optical attenuator before it is fed into the OEIC receiver by a multi-mode optical fiber.

A twin-output internally synchronized Agilent 81134A generator is used to provide separated clock signals for the bit pattern generator and the OEIC receiver with tuneable delay. For the measurement of the recovered signal, a Picoprobe 34A on-wafer active probe with 0.1 pF parallel 10 MΩ input impedance and an attenuation of 1:20 is used to deliver the signal to a 2 GHz bandwidth LeCroy WaveRunner 204Xi oscilloscope for eye diagram measurements, and to a Sympuls SBF-10G bit error analyser via a MiniCircuits ZFL-1000LN+ amplifier to obtain BER information. Finally, the average power of the modulated light signal is measured directly by the reading of the current flowing through the substrate of the OEIC (the anode of the integrated *pin* PD is formed by the p+ substrate and all transistors are isolated from the substrate by deep n-wells or n-wells), taking into account the sensitivity of the PD, which is 0.51 A W^−1^ for 635 nm and 0.53 A W^−1^ for 675 nm.

**Figure 4:**
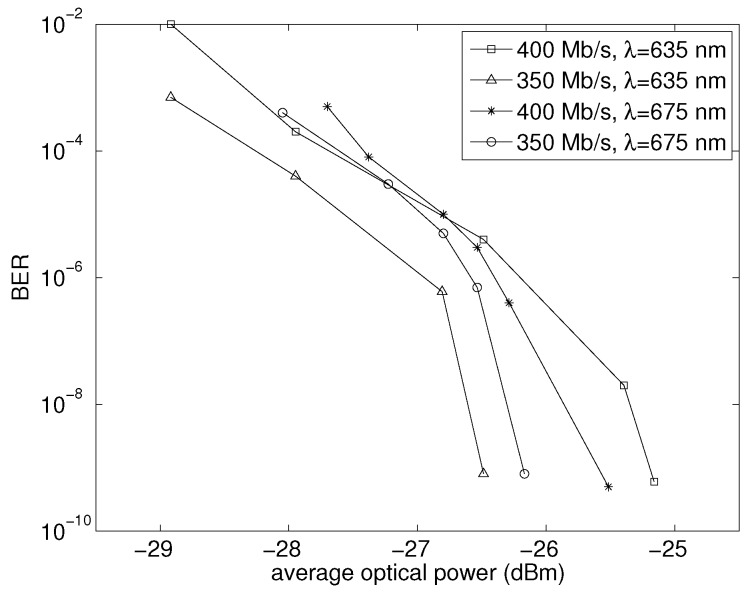
Sensitivity (corrected for ER = *∞*) at 350 Mbit/s and 400 Mbit/s for *λ* = 635 nm, extinction ratio = 10 and *λ* = 675 nm, extinction ratio = 5.5. 2^31^ − 1 PRBS.

**Figure 5:**
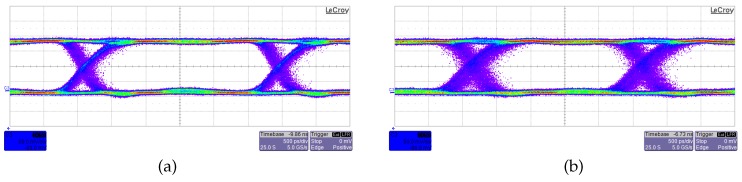
Eye diagram of the output signal of the OEIC at (**a**) 350 Mbit/s, average optical power −27.4 dBm; and (**b**) 400 Mbit/s, average optical power −26.0 dBm. 2^31^ − 1 PRBS, BER = 10^−9^, *λ* = 635 nm, extinction ratio = 10.

Figure 4 shows the results of the sensitivity measurements carried out on the OEIC. The receiver has been tested for the two laser sources, at 635 nm and 675 nm, respectively, for pseudo-random bit sequences of 2^31^ − 1 length at data rates of 400 Mbit/s and 350 Mbit/s. At 400 Mbit/s, the fabricated optical receiver shows a sensitivity equal to −26.0 dBm (corrected for ER = *∞*) for *λ* = 635 nm and −25.5 dBm for *λ* = 675 nm; in turn, at 350 Mbit/s, the sensitivity is -27.4 dBm for *λ* = 635 nm and −26.2 dBm for *λ* = 675 nm, all of them obtained at BER = 10^−9^. Figure 5 represents the measured eye diagrams for a 2^31^ − 1 PRBS with BER = 10^−9^. Figure 5a has been obtained at a data rate of 350 Mbit/s whereas Figure 5b at 400 Mbit/s.

The measured OEIC power consumption is 5.6 mW (1.7 mA from a 3.3 V power supply), of which only 790 μW are consumed by the regenerative latch while the remaining 4.8 mW are used by the auxiliary input clock and output buffers, and by the compensation current circuit. At 400 Mbit/s, the 790 μW consumed by the regenerative latch translates to an energy efficiency of 2 pJ/bit.

## 4. Comparison and Conclusions

A synchronous OEIC integrating receiver is presented in this paper, which is fabricated in an ASIC 0.35 μm CMOS technology with integrated *pin* PDs. Thanks to the reduced capacitance of the PDs, which is roughly 50 fF, a digital approach can be used for the receiver, eliminating the need of a high impedance TIA that adds chip area, without compromising the overall sensitivity of the receiver. In [3], a TIA + limiting amplifier in a feedback loop in an advanced 32 nm SOI CMOS achieved an energy efficiency of 1 pJ/bit, but needing an input optical average power of −1.7 dBm; on the other hand, in [5] a TIA with post amplifiers in 0.35 μm high-voltage CMOS achieved −31.8 dBm sensitivity, but requiring 55 pJ/bit to achieve a digital output, whereas the latch described here operates at only 2 pJ/bit. Excluding the PDs, those receivers occupy, respectively, 10,032 μm^2^ and 79,300 μm^2^, which contrast with the 1500 μm^2^ used by the latch plus one buffer stage at each output described here.

The OEIC shows a sensitivity of −26.0 dBm for a 2^31^ − 1 PRBS at 400 Mbit/s and −27.4 dBm at 350 Mbit/s, both for 635 nm and BER = 10^−9^. The comparison to other published integrating optical receivers, due to their digital operation, is strongly technology dependent in terms of speed when scaled to shorter nodes; in particular [8,9], achieve gigabit operation (1 Gbit/s and 1.2 Gbit/s) thanks to their implementation in 250 nm and 90 nm CMOS, whereas [12] operates at 320 Mbit/s and [7] at 180 Mbit/s using, respectively, 0.8 μm and 0.7 μm CMOS. In terms of sensitivity, the fabricated prototype achieves a 9.5 dB improvement over [8], 6.6 dB over [9], 9.4 dB over [12] in single-beam operation, and 14 dB over [7]. For its part, the receiver in [6] achieves a high −29.0 dBm sensitivity in a 0.35 μm CMOS process, but at a bit rate of just 5 Mbit/s. The good sensitivity of [6] results from the use of two regenerative feedbacks in the latch whereas we apply only one.

This structure has been used in many recent implementations of ORGAs, which incorporate an array of identical detecting cells with a spacing, both vertical and horizontal, of 90 μm in 0.35 μm CMOS [13], which can be reduced to 30 μm using a more advanced 180 nm CMOS technology [14]. In terms of the performance of the photodiodes, the former achieves a 10 ns response time with a sensitivity of −5.0 dBm using (25.5 μm)^2^ PDs, whereas the latter halves the response time to 5 ns with a slightly worse sensitivity of −4.5 dBm using (4.4 μm)^2^ PDs. Therefore, the suggested receiver achieves a large speed-up as well as eases the adjustment/coupling of the light input with much larger photodiodes considerably and therefore can be very advantageously integrated in ORGAs since it was shown that the implementation of a *pin* photodiode does not change the transistor parameters [15].

It has to be noted that the *pin* photodiode is responsible for the improved sensitivity without compromising the low-power characteristics of integrating optical receivers with an energy efficiency of 2 pJ/bit and at a reduced latch area of 50μ × 30 μm. This contrasts with that of large-area TIA-based receivers, typically at several hundreds of μm per side.
